# The Association Between the Maternal Pre-pregnancy Platelet Count and Fecundability in Mainland China: A Population-based Cohort Study

**DOI:** 10.2188/jea.JE20230191

**Published:** 2024-07-05

**Authors:** Xiaoyue Zhu, Jun Zhao, Xiang Hong, Yue Zhang, Xueying Yang, Hongguang Zhang, Rong Zhang, Yuanyuan Wang, Yan Xuan, Zuoqi Peng, Ya Zhang, Qiaomei Wang, Haiping Shen, Yiping Zhang, Donghai Yan, Xu Ma, Bei Wang

**Affiliations:** 1 Key Laboratory of Environment Medicine and Engineering of Ministry of Education, Department of Epidemiology and Health Statistics, School of Public Health, Southeast University, Nanjing, China; 2 Shanghai Key Laboratory of Sleep Disordered Breathing, Department of Otolaryngology Head and Neck Surgery, Shanghai Jiao Tong University Affiliated Sixth People’s Hospital, Shanghai, China; 3 National Research Institute for Family Planning, Beijing, China; 4 National Human Genetic Resources Center, Beijing, China; 5 Department of Maternal and Child Health, National Health Commission of the People’s Republic of China, Beijing, China

**Keywords:** fecundability, platelet count, pre-pregnancy, time to pregnancy

## Abstract

**Background:**

Currently, awareness about platelet count (PC) and its consequences for perinatal outcome have increased, but there is little reliable evidence on fecundability.

**Methods:**

Based on the National Free Pre-conception Check-up Projects supported by the Chinese government, 5,524,886 couples met the inclusion criteria and were included in this cohort study. Cox regression models were adopted to estimate fecundability ratios (FRs) and their 95% confidence intervals (CIs) for pre-pregnancy PC quintiles. Restricted cubic splines were used to flexibly model and visualize the relationship of PC with FRs. Microsoft SQL server and R software were used for data management and analysis.

**Results:**

The median of pre-pregnancy PC among women was 221.00 × 10^9^/L. The first (<177.00 × 10^9^/L) and second quintile (177.00–207.99 × 10^9^/L) of PC showed slightly increased fecundability (Q1: adjusted FR 1.05; 95% CI, 1.04–1.06; Q2: adjusted FR 1.04; 95% CI, 1.03–1.05), while higher quintals (Q4: 236.00–271.99 × 10^9^/L; Q5: ≥272.00 × 10^9^/L) were related to reduction of fecundability, when compared with the third quintile of PC (208.00–235.99 × 10^9^/L) (Q4: adjusted FR 0.96; 95% CI, 0.95–0.97; Q5: adjusted FR 0.88; 95% CI, 0.87–0.89). In the first quintiles (<177.00 × 10^9^/L), only 20.93% women had PC below 129.94 × 10^9^/L. An inverse-U-shaped association was consistently observed among women such that the lower PC within the normal range (<118.03 × 10^9^/L) and higher PC (>223.06 × 10^9^/L) were associated with the risk of reduced female fecundability (*P* for non-linearity < 0.01).

**Conclusion:**

PC is associated with female fecundability. Further classification of PC levels may deepen our understanding of the early warnings and significance of female fecundability.

## INTRODUCTION

Infertility is a highly prevalent condition globally and is characterized by the failure to establish a clinical pregnancy after 12 months of regular and unprotected sexual intercourse. Infertility affects approximately 15% of couples worldwide.^1^^–^^3^ It is estimated that more than 186 million people suffer from infertility.^4^ The prevalence of infertility was 25% among Chinese women who attempted pregnancy.^5^ Except for some common factors responsible for female infertility and unexplained infertility that have yet to be explicitly elucidated, the majority remain undiscovered.^2^

Remarkably, it has been recognized that pregnancy is associated with physiological and pathological changes in platelet count (PC) and function, which can be of clinical concern for underlying pregnancy-related disorder.^6^^–^^10^ In the fields of obstetrics and gynecology, evidence suggests a pertinent role for PC in physiological processes.^10^^,^^11^ Previous studies have shown that PC can be used as a reliable biomarker for early assessment of low-risk persistent gestational trophoblastic disease.^12^ PC is also related to platelet reactivity and aggregation.^13^^,^^14^ Several studies have recognized the clinical and diagnostic relevance of platelet hyperaggregability or thrombocythemia in women with a history of infertility and pregnancy loss.^8^^,^^15^ Moreover, epidemiological studies have suggested that PC is associated with neonatal birth weight and adverse perinatal outcomes.^16^^–^^18^

Many studies have emphasized the course of PC during pregnancy or correlation between abnormal PC and adverse outcomes in some patient groups. However, few have focused on the evidence concerning the relationship between PC and fecundability among childbearing-age women from general population.^7^^,^^8^^,^^19^ PC is known to be very stable over time in healthy individuals, while inter-individual variation in PC is considerable.^20^^–^^22^ To date, little is known about the effects of PC on female fecundability. Given the knowledge gap regarding natural female fecundability associated with pre-pregnancy PC and the need to further investigate the role of PCs in normal women who are prepared for pregnancy, we used a register-based cohort from the National Free Pre-conception Check-up Projects (NFPCP) to assess the potential impact of maternal pre-pregnancy PC on female fecundability.

## METHODS

### Study population and study design

**Figure 1.**
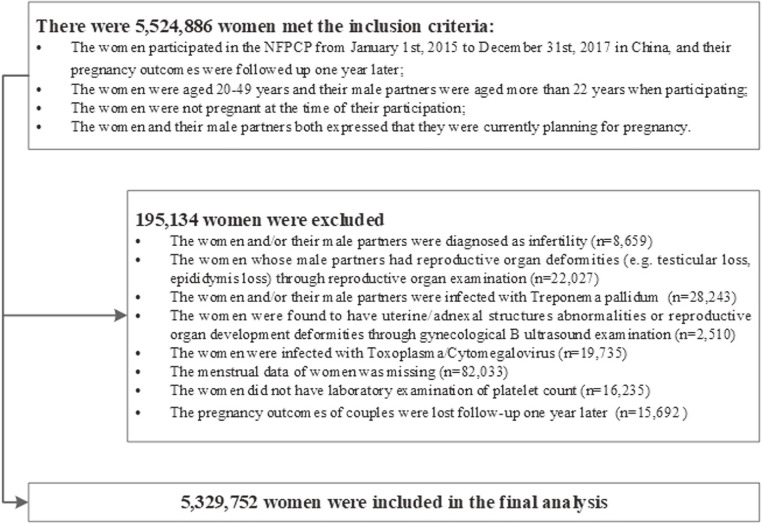
Flowchart of the study population. NFPCP, National Free Pre-conception Check-up Projects.

The NFPCP was a project supported by the Chinese government and aimed to provide free pre-pregnancy health examination and track follow-up of pregnancy outcomes routinely to reduce the incidence of adverse pregnancy outcomes for couples who planned to be pregnant.^23^^,^^24^ Detailed information regarding the protocols, original design, organization, and implementation had been described previously.^25^^–^^27^ Using the NFPCP database, we conducted a nationwide population-based cohort study to explore the association between PC distribution and fecundability in women of reproductive age in mainland China. A total of 5,524,886 couples who met the inclusion criteria were recruited in the present study (Figure 1).

### Data collection and operational definitions

All enrolled couples completed a standardized health questionnaire administered by experienced local health workers at baseline through face-to-face interviews. Gynecologic ultrasonography examination was performed to determine the presence of an existent gestational sac or deformities of the reproductive organs. Baseline information included women’ demographic characteristics and women’ health status and lifestyles. These characteristics were further categorized as follows: women’s age (20–24, 25–29, 30–34, 35–39, or ≥40 years old), region^28^ (Eastern, Central, or Western), ethnicity (Han or others), educational level (high school or below or bachelor degree or above), occupation (farmer, worker, civil servant, or others), pregnancy history (no or yes), number of children in current family (0, 1, or ≥2), alcohol consumption (no or yes). Contraception measures ever used was defined as the use of intrauterine devices, oral contraceptives, condoms, spermicide, or other contraceptives by oneself or spouse prior to the examination, regardless of duration. Menstrual status of female participants was obtained at baseline through questionnaires, and gynecological ultrasound examinations were performed in hospitals to identify gynecological abnormalities (endometriosis, polycystic ovarian syndrome, myoma, adenomyosis, adnexitis, uterine cavity adhesions, endometrial polyps, ovarian cysts, tubal dropsy, pelvic encapsulated effusion, and menorrhagia). Menstrual cycle regularity was grouped as follows: regular menstruation (menstrual cycle with an intermenstrual interval of 21–35 days, and the variation of cycle length from one period to another was ≤7 days^29^) and irregular menstruation. Tobacco exposure was divided into no or yes (active or passive smoker^30^). Health professionals measured the body weights (nearest 0.1 kg) and heights (nearest 0.1 cm) with coats, shoes and accessories removed according to the NFPCP’s standard protocols.^31^ Body mass index (BMI) was defined according to the criteria of the working group on obesity in China: underweight (BMI <18.5 kg/m^2^), normal (BMI 18.5–23.9 kg/m^2^), overweight (BMI 24–27.9 kg/m^2^) and obesity (BMI ≥28.0 kg/m^2^).^32^ Women’s systolic blood pressure (SBP) and diastolic blood pressure (DBP) were measured using electronic sphygmomanometers. Hypertension was defined as a SBP ≥140 mm Hg and/or a DBP ≥90 mm Hg.^33^ Fasting plasma glucose (FPG) levels were measured using a glucose oxidase assay. FPG level was further divided according to the World Health Organization guidelines^34^: diabetes (FPG ≥7.0 mmol/L), impaired fasting glucose (FPG ≥6.1 mmol/L) and normal (FPG <6.1 mmol/L). PC and hemoglobin concentrations were measured using laboratory examinations in accordance with the National Guide to Clinical Laboratory Procedures. Based on the World Health Organization criteria, measured Hb concentrations were adjusted for altitude level and smoking status.^35^ According to the anemia cut-off values recommended by the World Health Organization criteria, anemia in non-pregnant women aged 15 years and above was defined as a hemoglobin concentrations <120 g/L at sea level.^35^^,^^36^ The accuracy and stability of PC measurements and other laboratory tests were ensured by establishing a quality assurance system for the NFPCP.^24^

### Ascertainment of outcome

Follow-up interviews were routinely conducted by trained local health professionals via telephone after registration of the women recruited into the project. Follow-up interviews were conducted every 3 months to track the pregnancy status until they were confirmed to be pregnant or followed up for up to 1 year. The outcome was clinical pregnancy within 1 year, which was self-reported by women that was confirmed by a hospital pelvic ultrasound scan. Time to pregnancy (TTP) was defined as the interval between the last menstruation date (for pregnant couples) or the last follow-up date (for non-pregnant couples) obtained in last follow-up and the last menstruation date that obtained at baseline.^37^ TTP in months was calculated by TTP/30 among all women in this study. TTP in cycles for each woman was calculated separately by dividing the TTP by the average menstrual cycle length of her last 6 cycles regular menstruation among women with regular menstruation. TTP in months and TTP in cycles were both rounded downward.

### Statistical analysis

Descriptive statistics were used to present participant characteristics across the quintile distribution of baseline PC levels. One-way analysis of variance was used for continuous variables, and the χ^2^ test was used for categorical variables. The TTP in months was used for the main analyses. The Kaplan-Meier method was used to estimate the non-pregnancy rate, and the log-rank test was applied to detect differences among the PC categories. The Cox regression models for discrete survival time were adopted to estimate the fecundability ratios (FRs) and 95% confidence intervals (CIs). As the median PC was in the third quintile, women in the third quintile served as the reference group. Multivariate models were sequentially applied and adjusted for potential influencing factors for fecundability. Model 1 was adjusted for women’s demographic characteristics. In model 2, in addition to the factors included in model 1, we adjusted for women’s health status and lifestyles. FRs <1.00 indicate decreased fecundability, while FRs >1.00 denote enhanced fecundability.^38^ We used restricted cubic splines with four knots to visualize the relationship between maternal pre-pregnancy PC (modeled as a continuous variable) and fecundability.^39^ Subgroup analyses were performed by dividing the female baseline characteristics. Sensitivity analyses were performed to assess the robustness of the findings. First, in addition to the main analyses using TTP in months, TTP in cycles was calculated for women with regular menstruation. Second, we used multivariate imputation to impute the missing covariates and analyzed the imputed data for comparison with the original (total missingness) data. The covariate missingness in our study ranged from 0% to 3.62%. We created five imputed datasets using the multivariate imputation by chained equations (MICE) package in R software (R Foundation for Statistical Computing, Vienna, Austria), and the main analysis results were pooled after appropriate transformation.^40^^,^^41^ Third, we re-examined the FRs after excluding participants with excessively low (<100 × 10^9^/L) and high PC (>400 × 10^9^/L), which were considered abnormal PC values,^20^^,^^22^ to ensure that the results were consistent across both datasets. *E*-value was calculated for FR, and indicated how strong an unmeasured confounder would have to be to explain away an observed exposure-outcome relationship.^42^ Hosmer-Lemeshow goodness-of-fit tests were used to assess model fit.

The NFPCP Medical Service Information System was used for data collection. The Microsoft SQL Server 2012 (Microsoft Corporation, Redmond, WA, USA) was used for data management. All statistical analyses were performed using R (version 4.1.1). Two-sided *P*-values <0.05 were considered statistically significant.

### Ethical approval

This study was reviewed and approved by the Institutional Research Review Board of the National Health Commission and the National Health Council’s Ethics Review Committee. Informed consent was obtained from every subject before they participated in the study.

## RESULTS

**Figure 2.**
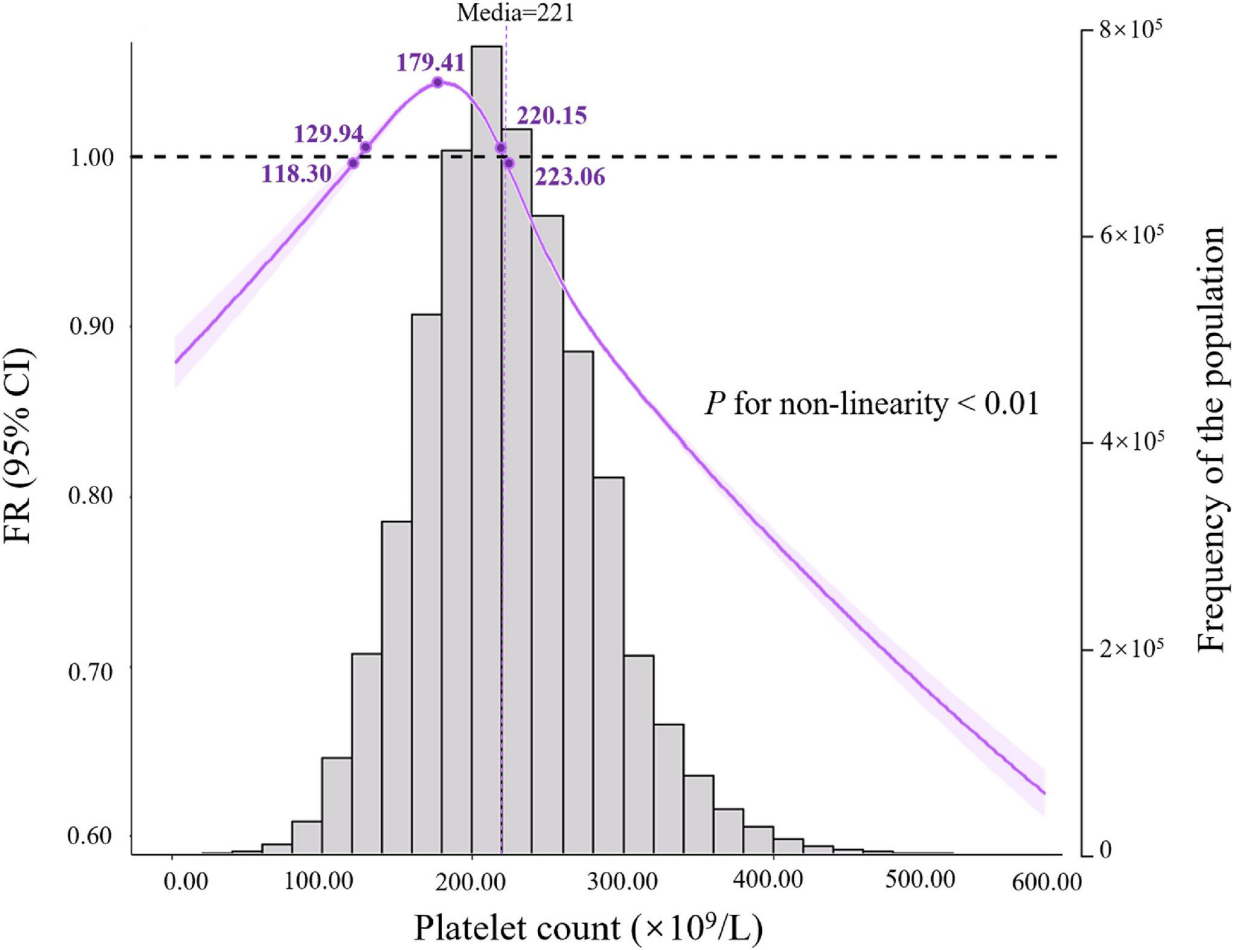
The association between platelet count and fecundability among women planning pregnancy fitted with the use of restricted cubic splines. There are 4 knots located at the 5th, 35th, 65th and 95th percentiles of the distribution of platelet count. Dashed lines correspond to reference values. Shaded areas represent 95% confidence intervals for FR. The median of platelet count was used as a reference. Histograms present distributions of platelet count in study participants. CI, confidence interval; FR, fecundability ratio. ^*^FRs were adjusted for women’s demographic characteristics (women’s age (continuous), husbands’ age (continuous), region, ethnicity, educational level, occupation, pregnancy history, number of children in the current family, age at menarche (continuous), menstrual cycle regularity) and women’s health status and lifestyles (body mass index (continuous), hypertension, fasting plasma glucose level, hemoglobin (continuous), alcohol consumption, tobacco exposure and contraceptive measures used before, and gynecological abnormalities).

After excluding 195,134 women according to the exclusion criteria, 5,329,752 women were included in the final analysis (Figure 1). In the current study, the overall mean and standard deviation of PC in all included women was 225.30 × 10^9^/L (59.48 × 10^9^/L), with median of 221 × 10^9^/L. The majority of women (98.41%) had a PC of 100.00–400.00 × 10^9^/L (Figure 2). The average ages were 28.07 (standard deviation [SD], 5.21) and 29.77 (SD, 5.55) years for the women and their husbands, respectively. Table 1 depicts the baseline characteristics of the study population by quintile of baseline pre-pregnancy PC levels. Although these baseline characteristics showed statistically significant differences among the five groups (*P* < 0.01), some of these groups had very small tiny differences, which may be due to the large sample size (Table 1).

**Table 1.** Baseline characteristics of women by quintile of platelet count

Variables	PC Q1 (<177.00 × 10^9^/L)	PC Q2 (177.00–207.99 × 10^9^/L)	PC Q3 (208.00–235.99 × 10^9^/L)	PC Q4 (236.00–271.99 × 10^9^/L)	PC Q5 (≥272.00 × 10^9^/L)	Missing, *n*	F/χ^2^	*P*
***N***	1,063,257	1,060,250	1,057,408	1,077,016	1,071,821			
**Women’s demographic characteristics**								
**Women’s age, years, mean (SD)**	28.09 (5.24)	27.98 (5.10)	28.02 (5.13)	28.05 (5.19)	28.23 (5.39)	0	362.50	<0.01
**Women’s age, years, *n* (%)**						0	3,064.20	<0.01
20–24	270,038 (25.40)	265,596 (25.05)	264,152 (24.98)	270,389 (25.11)	270,137 (25.20)			
25–29	469,800 (44.18)	482,994 (45.55)	478,874 (45.29)	482,636 (44.81)	462,258 (43.13)			
30–34	190,207 (17.89)	187,930 (17.73)	188,842 (17.86)	191,940 (17.82)	194,264 (18.12)			
35–39	88,222 (8.30)	84,045 (7.93)	84,976 (8.04)	88,724 (8.24)	95,501 (8.91)			
40–49	44,990 (4.23)	39,685 (3.74)	40,564 (3.84)	43,327 (4.02)	49,661 (4.63)			
**Husband’s age, years, mean (SD)**	28.00 (5.63)	28.00 (5.45)	28.00 (5.46)	28.00 (5.51)	29.00 (5.67)	0	559.70	<0.01
**Region, *n* (%)**								
Eastern	205,147 (19.29)	285,255 (26.91)	326,974 (30.92)	369,232 (34.28)	420,602 (39.24)	0	13,0133.00	<0.01
Central	558,736 (52.55)	550,311 (51.90)	519,158 (49.10)	492,601 (45.74)	420,073 (39.19)			
Western	299,374 (28.16)	224,684 (21.19)	211,276 (19.98)	215,183 (19.98)	231,146 (21.57)			
**Ethnicity, *n* (%)**								
Han	971,522 (92.27)	969,664 (92.47)	962,184 (92.03)	972,885 (91.41)	948,599 (89.60)	59,648	7,384.40	<0.01
Others	81,383 (7.73)	78,971 (7.53)	83,338 (7.97)	91,478 (8.59)	110,080 (10.40)			
**Educational level, *n* (%)**								
High school or below	825,329 (80.22)	799,606 (77.88)	796,589 (77.63)	808,862 (77.42)	806,472 (77.69)	165,344	3,241.10	<0.01
Bachelor degree or above	203,493 (19.78)	227,059 (22.12)	229,516 (22.37)	235,869 (22.58)	231,613 (22.31)			
**Occupation, *n* (%)**								
Farmer	711,191 (69.57)	699,827 (68.50)	698,138 (68.35)	708,905 (68.20)	703,450 (68.16)	192,757	4,040.60	<0.01
Worker	75,638 (7.40)	78,339 (7.67)	80,305 (7.86)	83,796 (8.06)	84,641 (8.20)			
Civil servant	93,423 (9.14)	103,999 (10.18)	107,403 (10.51)	112,784 (10.85)	114,802 (11.12)			
Others	142,038 (13.89)	139,451 (13.65)	135,638 (13.28)	134,014 (12.89)	129,213 (12.52)			
**Pregnancy history, *n* (%)**								
No	429,352 (40.41)	446,186 (42.11)	441,815 (41.81)	444,902 (41.34)	421,797 (39.38)	3,328	2,197.60	<0.01
Yes	633,232 (59.59)	613,359 (57.89)	614,971 (58.19)	631,416 (58.66)	649,394 (60.62)			
**Number of children in the current family, *n* (%)**								
0	469,327 (45.00)	487,247 (46.83)	482,250 (46.46)	486,499 (46.01)	464,500 (44.11)	98,212	3,325.90	<0.01
1	563,839 (54.06)	544,578 (52.34)	546,478 (52.65)	560,386 (53.00)	574,760 (54.58)			
≥2	9,733 (0.93)	8,603 (0.83)	9,162 (0.88)	10,472 (0.99)	13,706 (1.30)			
**Age at menarche, years, mean (SD)**	13.68 (1.18)	13.68 (1.18)	13.70 (1.17)	13.71 (1.18)	13.73 (1.20)	11,032	261.40	<0.01
**Menstrual cycle regularity, *n* (%)**								
Regular menstruation	1,026,742 (96.57)	1,023,806 (96.56)	1,020,823 (96.54)	1,037,945 (96.37)	1,030,833 (96.18)	0	358.68	<0.01
Irregular menstruation	36,515 (3.43)	36,444 (3.44)	36,585 (3.46)	39,071 (3.63)	40,988 (3.82)			
**Women’s health status and lifestyles**								
**BMI, kg/m^2^, mean (SD)**	21.27 (2.81)	21.38 (2.86)	21.55 (2.96)	21.75 (3.07)	22.20 (3.36)	10,704	261.40	<0.01
**BMI, kg/m^2^, *n* (%)**								
Underweight (<18.5)	145,915 (13.74)	139,512 (13.18)	129,500 (12.27)	121,999 (11.35)	108,255 (10.12)	10,704	57,453.00	<0.01
Normal (18.5–23.9)	763,549 (71.92)	757,463 (71.57)	744,500 (70.55)	744,401 (69.28)	697,262 (65.21)			
Overweight (24.0–27.9)	126,503 (11.91)	132,650 (12.53)	146,508 (13.88)	164,768 (15.33)	198,271 (18.54)			
Obesity (≥28.0)	25,762 (2.43)	28,704 (2.71)	34,737 (3.29)	43,379 (4.04)	65,410 (6.12)			
**Hypertension, *n* (%)**								
No	1,040,917 (98.34)	1,038,665 (98.39)	1,033,809 (98.21)	1,050,608 (97.99)	1,036,700 (97.19)	24,163	5,319.70	<0.01
Yes	17,522 (1.66)	16,951 (1.61)	18,858 (1.79)	21,597 (2.01)	29,962 (2.81)			
**Fasting plasma glucose level, *n* (%)**								
Normal	1,019,893 (96.38)	1,015,338 (96.23)	1,009,368 (95.95)	1,024,204 (95.58)	1,007,623 (94.49)	26,506	6,070.40	<0.01
Impaired fasting glucose	27,685 (2.62)	28,548 (2.71)	30,603 (2.91)	33,466 (3.12)	40,758 (3.82)			
Diabetes	10,604 (1.00)	11,204 (1.06)	12,050 (1.15)	13,920 (1.30)	17,982 (1.69)			
**Hemoglobin, g/L, mean (SD)**	128.51 (14.78)	128.94 (12.72)	129.42 (12.66)	129.65 (13.07)	128.22 (15.02)	1,056	9.29	<0.01
**Anemia, *n* (%)**								
No	819,440 (77.09)	849,615 (80.15)	861,603 (81.50)	880,038 (81.73)	827,717 (77.24)	1,056	13,292.00	<0.01
Yes	243,574 (22.91)	210,466 (19.85)	195,625 (18.50)	196,773 (18.27)	243,845 (22.76)			
**Alcohol consumption, *n* (%)**								
No	1,037,648 (97.83)	1,031,422 (97.54)	1,026,035 (97.26)	1,041,988 (96.98)	1,032,737 (96.58)	12,970	3,698.10	<0.01
Yes	23,034 (2.17)	26,064 (2.46)	28,881 (2.74)	32,430 (3.02)	36,543 (3.42)			
**Tobacco exposure, *n* (%)**								
No	960,828 (90.60)	952,520 (90.09)	947,712 (89.86)	961,336 (89.49)	953,136 (89.16)	13,963	1,434.70	<0.01
Yes	99,656 (9.40)	104,820 (9.91)	106,961 (10.14)	112,889 (10.51)	115,908 (10.84)			
**Contraceptive measures used before, *n* (%)**								
No	686,263 (64.75)	682,632 (64.60)	674,932 (64.03)	684,291 (63.75)	662,832 (62.05)	17,364	2,154.10	<0.01
Yes	373,575 (35.25)	374,108 (35.40)	379,206 (35.97)	389,080 (36.25)	405,469 (37.95)			
**Gynecological abnormalities, *n* (%)^a^**								
No	501,572 (93.62)	486,496 (93.54)	489,738 (93.38)	502,104 (93.16)	506,666 (92.42)	2,662,239	793.26	<0.01
Yes	34,202 (6.38)	33,622 (6.46)	34,700 (6.62)	36,866 (6.84)	41,547 (7.58)			

BMI, body mass index; PC, platelet count; Q, quintile; SD, standard deviation.

^a^Endometriosis, polycystic ovarian syndrome, myoma, adenomyosis, adnexitis, uterine cavity adhesions, endometrial polyps, ovarian cysts, tubal dropsy, pelvic encapsulated effusion, and menorrhagia were included.

Overall, 3,539,084 women (66.40%) had successfully conceived at the end of 1 year of follow-up. The second quintile of PC (177.00–207.99 × 10^9^/L) showed the significantly highest pregnancy rate (68.46%), while the fifth quintile (≥272.00 × 10^9^/L) had the lowest pregnancy rate (62.49%). The Kaplan-Meier plot showed a statistically significant difference among five PC categories (*P* for log-rank test <0.01) (eFigure 1). The results of the Hosmer-Lemeshow test showed that the model fit was acceptable (*P* > 0.05). Compared with the third quintile (208.00–235.99 × 10^9^/L) of PC group, the first (<177.00 × 10^9^/L) and the second quintiles (177.00–207.99 × 10^9^/L) of PC showed slightly increased fecundability (Q1: adjusted FR 1.05; 95% CI, 1.04–1.06; Q2: adjusted FR 1.04; 95% CI, 1.03–1.05). Compared to the third PC quintile, the women with the fourth (236.00–271.99 × 10^9^/L) and highest quintiles (≥272.00 × 10^9^/L) of PC had a 4% and 12% reduction in the probability of being pregnant in 1 year, respectively (Q4: adjusted FR 0.96; 95% CI, 0.95–0.97; Q5: adjusted FR 0.88; 95% CI, 0.87–0.89). The risk of subfecundity gradually increased with an increase level in the PC quintile (*P*_trend_ < 0.01) (Table 2).

**Table 2.** The fecundability ratios for women with different quintiles of baseline platelet count

Categories	Pregnancies/*N*	Pregnancy rate (%)	Crude FR (95% CI)	Adjusted FR (95% CI)^a^	Adjusted FR (95% CI)^b^
Q1 (<177.00 × 10^9^/L)	723,366/1,063,257	68.03	1.03 (1.02–1.04)	1.04 (1.03–1.05)	1.05 (1.04–1.06)
Q2 (177.00–207.99 × 10^9^/L)	725,852/1,060,250	68.46	1.04 (1.03–1.05)	1.04 (1.03–1.05)	1.04 (1.03–1.05)
Q3 (208.00–235.99 × 10^9^/L)	711,578/1,057,408	67.29	Ref	Ref	Ref
Q4 (236.00–271.99 × 10^9^/L)	708,481/1,077,016	65.78	0.94 (0.93–0.95)	0.95 (0.94–0.96)	0.96 (0.95–0.97)
Q5 (≥272.00 × 10^9^/L)	669,807/1,071,821	62.49	0.86 (0.85–0.87)	0.87 (0.86–0.88)	0.88 (0.87–0.89)
*P* for trend					<0.01

CI, confidence interval; FR, fecundability ratio; Q, quintile.

^a^FRs were adjusted for women’s demographic characteristics (women’s age (continuous), husbands’ age (continuous), region, ethnicity, educational level, occupation, pregnancy history, number of children in the current family, age at menarche (continuous) and menstrual cycle regularity).

^b^FRs were additionally adjusted for women’s health status and lifestyles (body mass index (continuous), hypertension, fasting plasma glucose level, hemoglobin (continuous), alcohol consumption, tobacco exposure, contraceptive measures used before, and gynecological abnormalities).

A right-skewed, inverse-U-shaped relationship between maternal pre-pregnancy PC and fecundability was detected using restricted cubic splines (*P* for non-linearity < 0.01). Increased FR was noted until a PC of approximately 179.41 × 10^9^/L, and FR started to decrease thereafter. The plot presented a statistical increase of fecundability within the PC range of 129.94–220.15 (×10^9^/L) and statistical decrease of fecundability below the PC range of 118.30 × 10^9^/L or above 223.06 × 10^9^/L, indicating that too lower or higher PC was associated with decreased fecundability (Figure 2). Except for the first quintile, the plot of restricted cubic splines was consistent with the findings of the categorical analysis. As in the first PC quintile (<177.00 × 10^9^/L) for women, only 222,528 (20.93%) women had PC below 129.94 × 10^9^/L; 87.75% had a PC above 118.30 × 10^9^/L in this quintile. Furthermore, additional categories were divided into four knots located at the 5th, 35th, 65th, and 95th percentiles of the PC distribution according to the restricted cubic splines. Categories 1 (<134.00 × 10^9^/L), 4 (244.00–326.99 × 10^9^/L) and 5 ( ⩾ 327.00 × 10^9^/L) showed decreased fecundability compared to Category 3 (200.00–243.99 × 10^9^/L) (Category 1: adjusted FR 0.98; 95% CI, 0.97–0.99; Category 4: adjusted FR 0.92; 95% CI, 0.91–0.93; Category 5: adjusted FR 0.81; 95% CI, 0.80–0.82). Only Category 2 (134.00–199.99 × 10^9^/L) showed increased fecundability when compared with Category 3 (adjusted FR 1.05; 95% CI, 1.04–1.07) (eTable 1).

**Figure 3.**
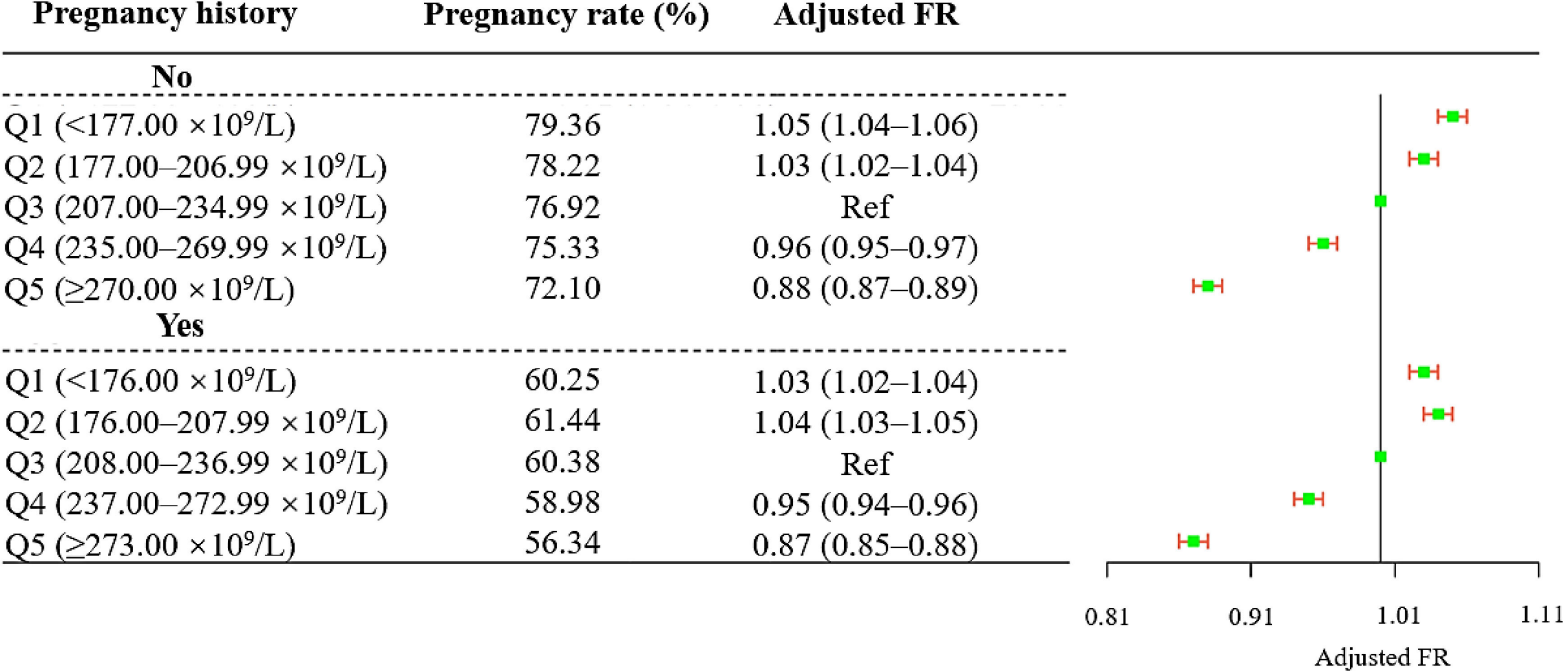
The association between platelet count and fecundability in an analysis stratified by pregnancy history. CI, confidence interval; FR, fecundability ratio; Q, quintile. ^*^FRs were adjusted for women’s demographic characteristics (women’s age (continuous), husbands’ age (continuous), region, ethnicity, educational level, occupation, number of children in the current family, age at menarche (continuous), menstrual cycle regularity) and women’s health status and lifestyles (body mass index (continuous), hypertension, fasting plasma glucose level, hemoglobin (continuous), alcohol consumption, tobacco exposure and contraceptive measures used before, and gynecological abnormalities).

**Figure 4.**
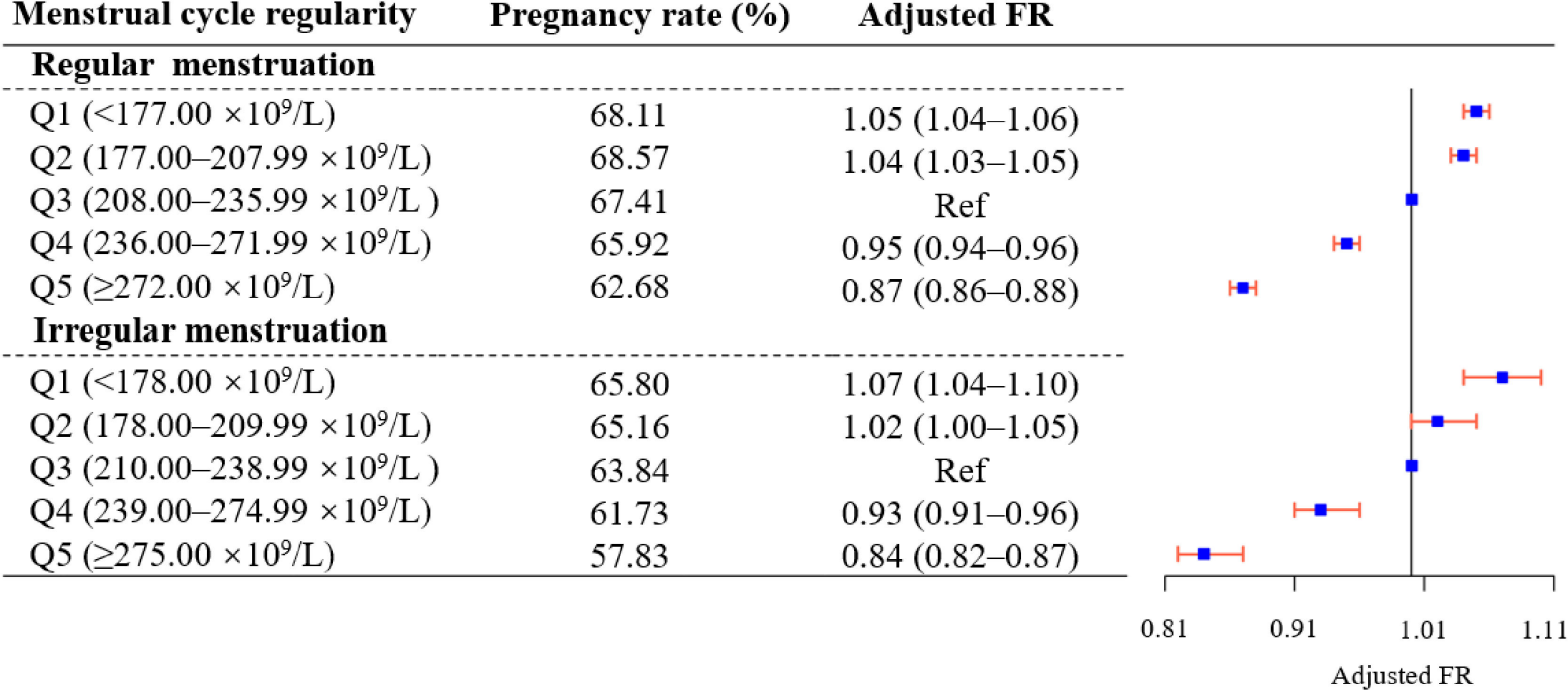
The association between platelet count and fecundability in an analysis stratified by menstrual cycle regularity. CI, confidence interval; FR, fecundability ratio; Q, quintile. ^*^FRs were adjusted for women’s demographic characteristics (women’s age (continuous), husbands’ age (continuous), region, ethnicity, educational level, occupation, pregnancy history, number of children in the current family, age at menarche (continuous)) and women’s health status and lifestyles (body mass index (continuous), hypertension, fasting plasma glucose level, hemoglobin (continuous), alcohol consumption, tobacco exposure and contraceptive measures used before, and gynecological abnormalities).

In the subgroup analysis stratified by pregnancy history, associations of pregnancy history-specific PC quintiles with fecundability showed similar patterns between women with or without pregnancy history (Figure 3; eTable 2). The spline curves stratified by women with or without pregnancy history presented different ‘unhealthy range’ of PC in different subgroups (eFigure 2). Additionally, the associations between PC and fecundability in women with regular or irregular menstruation were estimated. A similar association across the strata was found when compared with the third quintile (Figure 4; eTable 2). The FRs calculated using TTP in cycles among women with regular menstruation were similar to those calculated using the TTP in months (eTable 3). Spline curves separately stratified by menstrual cycle regularity showed that different cut points for decreased fecundability in women with regular or irregular menstruation, respectively (eFigure 3). Furthermore, results stratified by other characteristics, were broadly consistent across strata (eTable 2).

Moreover, the results of the imputed data were similar to those of the main outcomes (eTable 4). After removing women with excessively lower PC (<100 × 10^9^/L) (*n* = 44,721) and higher PC (>400 × 10^9^/L) (*n* = 39,855), the sensitivity analysis showed little difference in the estimated association between PC levels and female fecundability (eTable 5). The *E*-values were 1.22, 1.20, 1.17 for the first, second, and fourth quintile, respectively. E-value of the highest PC quintile was 1.41, indicating that the observed odds of 0.88 could be explained away by an unmeasured confounder associated with both PC level and female fecundability by and fecundability ratio of 1.41-fold.

## DISCUSSION

PC is associated with several underlying pregnancy-related disorder.^6^^–^^8^^,^^10^^,^^43^ However, evidence regarding PC among general women of childbearing age is sparse, especially studies on female fecundability. To the best of our knowledge, there are currently no studies with preconception enrollment of women whose PC was assessed and the association with fecundability was prospectively measured. Hence, it is clinically important to explore whether PC may have a potential ‘unhealthy range’ in terms of female fecundability. In this large cohort study with pre-pregnancy enrollment of women attempting to become pregnant, we suggested a role for PC in fecundability. Herein, we found that the first and second PC quintiles were statistically significantly associated with increased fecundability, while higher quintals were related to a reduction in fecundability, compared with the third PC quintile which contained the median of PC. It is worth noting that only 12.25% of women had a PC below 118.03 × 10^9^/L in the first quintile, which was considered to be a lower PC within the normal range. There was an inverse-U-shaped association among women such that the lower PC within the normal range (<118.03 × 10^9^/L) and higher PC (<223.06 × 10^9^/L) were associated with the risk of reduced fecundability, which could be considered as a ‘unhealthy range’ for female fecundability. Additionally, results of the associations stratified by other characteristics were broadly consistent across strata. Thus, the results can be considered accurate and robust.

Studies on thrombocytopenia in pregnancy have indicated that some causes of thrombocytopenia are serious medical disorders that have the potential for maternal and fetal morbidity,^17^^,^^18^ such as gestational thrombocytopenia, posing harm to maternal or fetal health.^9^^,^^44^^,^^45^ A previous study suggested that pregnant women with PC of less than 100 × 10^9^/L should undergo further clinical and laboratory assessment.^46^ During human pregnancy, maternal PC decreases gradually from the first to third trimester.^47^^,^^48^ Thus, it is useful to do examination the of PC before pregnancy. It seems that low PC not only has implications for maternal health in pregnancy, but also effects fecundability. In the present study, the first (<177.00 × 10^9^/L) and second PC quintiles (177.00–207.99 × 10^9^/L) showed slightly increased fecundability compared with the median group. However, it is notable that the lower PC within normal range (<118.30 × 10^9^/L) presented reduced fecundability. Until now, no study has revealed the association between PC and female fecundability. Therefore, there are currently no studies that directly support our findings. In addition to maintenance of PC within normal ranges, keeping PC within a ‘healthy range’ may be helpful for fecundability.

In terms of higher levels of PC, our study found that higher PC quintiles were related to reduced fecundability compared to the median PC group. A reduction in fecundability was found when PC was above 223.06 × 10^9^/L. A study based on the Born in Guangzhou Cohort Study suggested that higher PC quintiles are associated with increased risk for both early-onset preeclampsia/eclampsia (PC ≥252 × 10^9^/L) compared with the first quintile,^10^ which revealed that a higher PC may be harmful in pregnant women. Notably, it is demonstrated that PC is associated with platelet reactivity,^13^^,^^14^ and abnormal platelet aggregation plays a critical role in some adverse pregnancies.^11^ However, these studies focused only on infertile women, and the causal inference between PC and fecundability in healthy women could not be revealed. Although the mechanisms underlying the increased risk of PC associated with decreased fecundability are not fully understood, the potential implications of PC cannot be ignored.

Currently, little was known about the association of PC with fecundability in women in the general population. It is worth noting that we put emphasis on pre-pregnancy women from the general population, and 98.41% of the women in our study had PC ranging from 100.00 × 10^9^/L to 400.00 × 10^9^/L, in which most of them could be considered as having normal PC. Additionally, results of the sensitivity analysis reserving the normal range of PC and the subgroup analyses also presented similar outcome in our study, which assessed and ensured the robustness of our findings. In our study, we found that the normal PC range did not fully reflect the boundaries of fecundability, and further classification of PC levels may deepen the early warning and significance of female fecundability. In our study, an inverse-U-shaped association was found between PC and female fecundability, which may indicate a ‘unhealthy range’ of PC for fecundability status. Remarkably, low and high pre-pregnancy PC need to be focused on, though the proportion of women with too low PC levels in the first quintile was small.

Taken together, our study highlights the effect of PC in predicting female fecundability and suggests that attention should be paid to pre-pregnancy women with lower extreme or higher PC levels. Furthermore, a study that aimed to identify age- and sex-specific reference intervals for platelet count conducted in Italy suggested that the age-, sex-, and origin-related variability of platelet count was very wide. Therefore, it was appropriate to identify age- and sex-specific reference intervals of PC.^22^ Thus, characteristic-specific PC ranges were used to estimate the association of the subgroup analyses in our study. Therefore, it is more practical and appropriate to understand the distribution of PC in populations with different characteristics, reflect the boundaries that exactly match the specific range, and detect the fecundability association of different subgroups. More practically, measuring PC was relatively inexpensive. Our study suggests new relevant aspects of PC, and we propose that PC deviation could potentially be of value in estimating fecundability. Notably, small differences can be easily detected with larger sample sizes. Therefore, the clinical importance of these ‘significant’ differences should be treated with caution when interpreting the results.

This study has several merits. First, the large population-based sampling framework from 31 provinces in mainland China, standardized data collection methods, and strict laboratory quality control ensured reliability of the data. Second, as a prospective study, rigorous inclusion and exclusion criteria were implemented, in which women attempting pregnancy were eligible, so as to minimize the bias in this study, such as recall and response biases. Third, to minimize the potential confounding factors, several multivariate-adjusted analyses with different factors were considered. Moreover, several sensitivity analyses were conducted to improve the sensitivity and robustness of the results.

However, this study has some limitations. First, we lacked the time for participants to attempt pregnancy before enrollment, which would overestimate the fecundability. Conversely, some couples might suspend pregnancy plans during follow-up due to certain emergencies (which might be unreported during follow-up conducted every 3 months), which would underestimate the fecundability. Second, potential bias could not be avoided, as information on behavioral characteristics was self-reported. Unknown confounders cannot be avoided in observational studies. Third, owing to the lack of information on sperm quality and sexual frequency of couples, we could not adjust for these factors in the present study. Fourth, origin-related variability of PC is wide and is influenced by genetic background.^22^ Consequently, speculation on other racial and ethnic groups should be interpreted with caution as our study was limited to the Chinese population.

Overall, compared with the third PC quintile (208.00–235.99 × 10^9^/L), the first and second quintiles showed slightly increased fecundability, while higher quintiles were associated with reduced female fecundability. An inverse-U-shaped relationship was found between maternal pre-pregnancy PC and fecundability, indicating that too low PC within the normal range (<118.30 × 10^9^/L) and higher PC (>223.06 × 10^9^/L) were associated with reduced female fecundability risk. By stratifying PC into distinct classifications, it becomes possible to better understand the potential implications and significance of different PC levels on female fecundability.

## SUPPLEMENTARY MATERIAL

eTable 1. The association between platelet count and fecundability with the categories divided by restricted cubic splines

eTable 2. Subgroup analysis for the association between platelet count and fecundability

eTable 3. The fecundability ratios for women with regular menstruation that calculated by TTP in cycles

eTable 4. Sensitivity analysis for the association between platelet count and fecundability (based on the imputed data after multivariate imputation)

eTable 5. Sensitivity analysis for the association between platelet count and fecundability (excluding the women whose PLT < 100 × 10^9^/L or PLT > 400 × 10^9^/L)

eFigure 1. Kaplan-Meier plot for pre-pregnancy maternal platelet count status (n = 5,329,752)

eFigure 2. The association between platelet count and fecundability stratified by pregnant history with the use of restricted cubic splines

eFigure 3. The association between platelet count and fecundability stratified by menstrual cycle regularity with the use of restricted cubic splines
