# Supplementary material for: The Association Between the Maternal Pre-pregnancy Platelet Count and Fecundability in Mainland China: A Population-based Cohort Study
Source: J Epidemiol. 2024 Jul 5;34(7):340–8. doi: 10.2188/jea.JE20230191 (PMC11167265; doi:10.2188/jea.JE20230191)
Supplement: Supplementary file 1 [file je-34-340-s001.pdf]

eTable 1. The association between platelet count and fecundability with the categories divided by restricted cubic splines

Categories	Pregnancies/ N	Pregnancy rate (%)	Adjusted FR (95% CI) [a]
Category 1 (<134.00 $\times 10^9$/L)	169,485/ 259,987	65.19	0.98 (0.97–0.99)
Category 2 (134.00–199.99 $\times 10^9$/L)	1,071,626/ 1,557,376	68.81	1.05 (1.04–1.07)
Category 3 (200.00–243.99 $\times 10^9$/L)	1,095,093/ 1,629,021	67.22	Ref
Category 4 (244.00–326.99 $\times 10^9$/L)	1,041,343/ 1,611,876	64.60	0.92 (0.91–0.93)
Category 5 (≥327.00 $\times 10^9$/L)	161,537/ 271,492	59.50	0.81 (0.80–0.82)
P for trend			<0.01

CI, confidence interval; FR, fecundability odds ratio; Q, quintile.

There are 4 knots located at the 5th, 35th, 65th and 95th percentiles of the distribution of platelet count (134×10^9/L, 200×10^9/L, 244×10^9/L, 327×10^9/L).

[a] FRs were adjusted for women's demographic characteristics (women's age (continuous), husband's age (continuous), region, ethnicity, educational level, occupation, pregnancy history, number of children in the current family, age at menarche (continuous), menstrual cycle regularity) and women's health status and lifestyles (body mass index (continuous), hypertension, fasting plasma glucose level, hemoglobin (continuous), alcohol consumption, tobacco exposure, contraceptive measures used before, and gynecological abnormalities).

eTable 2. Subgroup analysis for the association between platelet count and fecundability

**Characteristics**	**Categories**	**Pregnancies/ N**	**Pregnancy rate (%)**	**Adjusted FR (95% CI)** [a]
**Women's age, years**				
20–24	Q1 (<176.00 ×10^9/L)	202,246/ 262,647	77.00	1.05 (1.04–1.06)
	Q2 (176.00–207.99 ×10^9/L)	209,528/ 272,987	76.75	1.04 (1.03–1.05)
	Q3 (208.00–235.99 ×10^9/L)	198,970/ 264,152	75.32	Ref
	Q4 (236.00–271.99 ×10^9/L)	199,180/ 270,389	73.66	0.95 (0.94–0.96)
	Q5 (≥272.00 ×10^9/L)	189,129/ 270,137	70.01	0.87 (0.86–0.88)
	Total	999,053/ 1,340,312	74.54	
	P for trend		<0.01	
25–29	Q1 (<177.00 ×10^9/L)	339,124/ 469,800	72.18	1.05 (1.04–1.06)
	Q2 (177.00–206.99 ×10^9/L)	335,799/ 466,116	72.04	1.04 (1.03–1.05)
	Q3 (207.00–234.99 ×10^9/L)	339,792/ 479,559	70.86	Ref
	Q4 (235.00–269.99 ×10^9/L)	329,497/ 475,542	69.29	0.95 (0.94–0.96)
	Q5 (≥270.00 ×10^9/L)	322,800/ 485,545	66.48	0.88 (0.87–0.89)
	Total	1,667,012/ 2,376,562	70.14	
	P for trend		<0.01	
30–34	Q1 (<177.00 ×10^9/L)	120,668/ 190,207	63.44	1.03 (1.02–1.04)
	Q2 (177.00–207.99 ×10^9/L)	119,991/ 187,930	63.85	1.03 (1.02–1.04)
	Q3 (208.00–235.99 ×10^9/L)	119,353/ 188,842	63.20	Ref
	Q4 (236.00–271.99 ×10^9/L)	118,952/ 191,940	61.97	0.97 (0.95–0.98)
	Q5 (≥272.00 ×10^9/L)	116,019/ 194,264	59.72	0.89 (0.88–0.90)
	Total	594,983/ 953,183	62.42	
	P for trend		<0.01	

35–39	Q1 (<177.00 ×10^9/L)	45,245/ 88,222	51.29	1.03 (1.01–1.05)
	Q2 (177.00–208.99 ×10^9/L)	46,124/ 86,952	53.05	1.04 (1.02–1.06)
	Q3 (209.00–237.99 ×10^9/L)	45,785/ 87,578	52.28	Ref
	Q4 (238.00–274.99 ×10^9/L)	45,615/ 88,881	51.32	0.96 (0.95–0.98)
	Q5 (≥275.00 ×10^9/L)	44,362/ 89,835	49.38	0.90 (0.88–0.92)
	Total	227,131/ 441,468	51.45	
	P for trend		<0.01	
40–49	Q1 (<175.00 ×10^9/L)	9,758/ 42,841	22.78	1.00 (0.96–1.02)
	Q2 (175.00–208.99 ×10^9/L)	10,661/ 43,237	24.66	1.02 (1.02–1.06)
	Q3 (209.00–239.99 ×10^9/L)	10,751/ 44,569	24.12	Ref
	Q4 (240.00–277.99 ×10^9/L)	10,131/ 43,511	23.28	0.98 (0.94–1.02)
	Q5 (≥278.00 ×10^9/L)	9,604/ 44,069	21.79	0.91 (0.88–0.95)
	Total	50,905/ 218,227	23.33	
	P for trend		<0.01	
**Region**				
Eastern	Q1 (<191.00 ×10^9/L)	214,441/ 315,733	67.92	1.09 (1.08–1.10)
	Q2 (191.00–219.99 ×10^9/L)	209,238/ 316,216	66.17	1.05 (1.04–1.06)
	Q3 (220.00–248.99 ×10^9/L)	212,570/ 329,185	64.57	Ref
	Q4 (249.00–283.99 ×10^9/L)	201,656/ 318,014	63.41	0.96 (0.95–0.97)
	Q5 (≥284.00 ×10^9/L)	195,749/ 328,062	59.67	0.86 (0.85–0.87)
	Total	1,033,654/ 1,607,210	64.31	
	P for trend		<0.01	
Central	Q1 (<173.00 ×10^9/L)	334,609/ 497,007	67.32	1.02 (1.01–1.03)
	Q2 (202.00–228.99 ×10^9/L)	353,828/ 504,881	70.08	1.05 (1.04–1.06)
	Q3 (229.00–263.99 ×10^9/L)	353,741/ 510,060	69.35	Ref

	Q4 (264.00–271.99 ×10^9/L)	347,864/ 512,967	67.81	0.94 (0.93–0.95)
	Q5 (≥264.00 ×10^9/L)	337,098/ 515,964	65.33	0.87 (0.86–0.88)
	Total	1,727,140/ 2,540,879	67.97	
	P for trend		<0.01	
Western	Q1 (<166.00 ×10^9/L)	160,053/ 233,874	68.44	1.06 (1.04–1.07)
	Q2 (166.00–200.99 ×10^9/L)	160,485/ 236,774	67.78	1.03 (1.02–1.05)
	Q3 (201.00–231.99 ×10^9/L)	156,784/ 236,769	66.22	Ref
	Q4 (232.00–269.99 ×10^9/L)	150,567/ 233,293	64.54	0.97 (0.96–0.98)
	Q5 (≥270.00 ×10^9/L)	150,401/ 240,953	62.42	0.92 (0.91–0.93)
	Total	778,290/ 1,181,663	65.86	
	P for trend		<0.01	
**Ethnicity**				
Han	Q1 (<176.00 ×10^9/L)	646,244/ 944,322	68.43	1.04 (1.03–1.05)
	Q2 (176.00–206.99 ×10^9/L)	664,037/ 963,049	68.95	1.04 (1.03–1.05)
	Q3 (207.00–234.99 ×10^9/L)	653,359/ 963,849	67.79	Ref
	Q4 (235.00–270.99 ×10^9/L)	650,879/ 982,829	66.23	0.95 (0.94–0.96)
	Q5 (≥271.00 ×10^9/L)	610,879/ 970,805	62.92	0.87 (0.86–0.88)
	Total	3,225,398/ 4,824,854	66.85	
	P for trend		<0.01	
Others	Q1 (<180.00 ×10^9/L)	55,218/ 87,584	63.05	1.07 (1.05–1.09)
	Q2 (180.00–212.99 ×10^9/L)	56,300/ 88,157	63.86	1.08 (1.06–1.10)
	Q3 (213.00–243.99 ×10^9/L)	55,810/ 90,110	61.94	Ref
	Q4 (244.00–281.99 ×10^9/L)	54,566/ 89,119	61.23	0.98 (0.96–0.99)
	Q5 (≥282 ×10^9/L)	53,592/ 90,280	59.36	0.89 (0.88–0.91)
	Total	275,486/ 445,250	61.87	

	P for trend		<0.01	
**Educational level**				
High school or below	Q1 (<176.00 ×10^9/L)	540,203/ 802,780	67.29	1.04 (1.03–1.05)
	Q2 (176.00–206.99 ×10^9/L)	542,508/ 794,428	68.29	1.04 (1.03–1.05)
	Q3 (207.00–235.99 ×10^9/L)	553,435/ 824,316	67.14	Ref
	Q4 (236.00–270.99 ×10^9/L)	518,686/ 790,188	65.64	0.95 (0.94–0.96)
	Q5 (≥271.00 ×10^9/L)	513,975/ 825,146	62.29	0.87 (0.86–0.88)
	Total	2,668,807/ 4,036,858	66.11	
	P for trend		<0.01	
Bachelor degree or above	Q1 (<180.00 ×10^9/L)	155,443/ 221,514	70.17	1.07 (1.06–1.09)
	Q2 (180.00–209.99 ×10^9/L)	155,145/ 225,194	68.89	1.03 (1.02–1.05)
	Q3 (210.00–237.99 ×10^9/L)	154,030/ 228,512	67.41	Ref
	Q4 (238.00–272.99 ×10^9/L)	148,933/ 225,668	66.00	0.97 (0.96–0.98)
	Q5 (≥273.00 ×10^9/L)	143,168/ 226,662	63.16	0.90 (0.89–0.91)
	Total	756,719/ 1,127,550	67.11	
	P for trend		<0.01	
**Pregnancy history**				
No	Q1 (<177.00 ×10^9/L)	340,732/ 429,352	79.36	1.05 (1.04–1.06)
	Q2 (177.00–206.99 ×10^9/L)	336,775/ 430,526	78.22	1.03 (1.02–1.04)
	Q3 (207.00–234.99 ×10^9/L)	340,819/ 443,064	76.92	Ref
	Q4 (235.00–269.99 ×10^9/L)	330,012/ 438,072	75.33	0.96 (0.95–0.97)
	Q5 (≥270.00 ×10^9/L)	319,426/ 443,038	72.10	0.88 (0.87–0.89)
	Total	1667,764/ 2,184,052	76.36	
	P for trend		<0.01	
Yes	Q1 (<176.00 ×10^9/L)	371,089/ 615,884	60.25	1.03 (1.02–1.04)

	Q2 (176.00–207.99 ×10^9/L)	387,506/ 630,707	61.44	1.04 (1.03–1.05)
	Q3 (208.00–236.99 ×10^9/L)	384,352/ 636,551	60.38	Ref
	Q4 (237.00–272.99 ×10^9/L)	367,528/ 623,097	58.98	0.95 (0.94–0.96)
	Q5 (≥273.00 ×10^9/L)	358,376/ 636,133	56.34	0.87 (0.86–0.88)
	Total	1,868,851/ 3,142,372	59.47	
	P for trend		<0.01	
**Occupation**				
Farmer	Q1 (<176.00 ×10^9/L)	478,532/ 691,108	69.24	1.03 (1.02–1.04)
	Q2 (176.00–206.99 ×10^9/L)	488,952/ 695,714	70.28	1.05 (1.04–1.06)
	Q3 (207.00–235.99 ×10^9/L)	497,669/ 722,334	68.90	Ref
	Q4 (236.00–270.99 ×10^9/L)	465,106/ 692,455	67.17	0.96 (0.95–0.97)
	Q5 (≥271.00 ×10^9/L)	457,840/ 719,900	63.60	0.87 (0.86–0.88)
	Total	2,388,099/ 3,521,511	67.81	
	P for trend		<0.01	
Worker	Q1 (<179.00 ×10^9/L)	51,289/ 79,885	64.20	1.03 (1.01–1.05)
	Q2 (179.00–209.99 ×10^9/L)	51,981/ 79,668	65.25	1.02 (0.99–1.04)
	Q3 (210.00–237.99 ×10^9/L)	52,089/ 80,006	65.11	Ref
	Q4 (238.00–273.99 ×10^9/L)	52,224/ 82,034	63.66	0.97 (0.96–0.99)
	Q5 (≥274.00 ×10^9/L)	49,654/ 81,126	61.21	0.93 (0.91–0.95)
	Total	257,237/ 402,719	63.88	
	P for trend		<0.01	
Civil servant	Q1 (<181.00 ×10^9/L)	69,113/ 104,834	65.93	1.05 (1.04–1.07)
	Q2 (181.00–210.99 ×10^9/L)	67,860/ 104,412	64.99	1.02 (1.01–1.04)
	Q3 (211.00–239.99 ×10^9/L)	70,267/ 109,965	63.90	Ref
	Q4 (240.00–274.99 ×10^9/L)	66,477/ 105,696	62.89	0.98 (0.96–0.99)

	Q5 (≥275.00 ×10^9/L)	64,743/ 107,504	60.22	0.91 (0.89–0.93)
	Total	338,460/ 532,411	63.57	
	P for trend		<0.01	
Others	Q1 (<175.00 ×10^9/L)	87,855/ 134,689	65.23	1.10 (1.08–1.12)
	Q2 (175.00–205.99 ×10^9/L)	87,852/ 136,680	64.28	1.04 (1.02–1.05)
	Q3 (206.00–232.99 ×10^9/L)	83,504/ 132,397	63.07	Ref
	Q4 (233.00–268.99 ×10^9/L)	86,335/ 138,537	62.32	0.98 (0.97–0.99)
	Q5 (≥269.00 ×10^9/L)	82,311/ 138,051	59.62	0.91 (0.90–0.93)
	Total	427,857/ 680,354	62.89	
	P for trend		<0.01	
**Number of children in the current family**				
0	Q1 (<177.00 ×10^9/L)	370,782/ 469,327	79.00	1.05 (1.04–1.06)
	Q2 (177.00–206.99 ×10^9/L)	366,017/ 470,164	77.85	1.03 (1.02–1.04)
	Q3 (207.00–234.99 ×10^9/L)	370,139/ 483,487	76.56	Ref
	Q4 (235.00–269.99 ×10^9/L)	359,075/ 479,183	74.93	0.96 (0.95–0.97)
	Q5 (≥270.00 ×10^9/L)	349,904/ 487,662	71.75	0.88 (0.87–0.89)
	Total	1,815,917/ 2,389,823	75.99	
	P for trend		<0.01	
1	Q1 (<176.00 ×10^9/L)	322,294/ 548,363	58.77	1.03 (1.02–1.04)
	Q2 (176.00–207.99 ×10^9/L)	336,941/ 560,054	60.16	1.05 (1.04–1.06)
	Q3 (208.00–235.99 ×10^9/L)	323,711/ 546,478	59.24	Ref
	Q4 (236.00–272.99 ×10^9/L)	331,238/ 572,127	57.90	0.95 (0.94–0.96)
	Q5 (≥273.00 ×10^9/L)	311,177/ 563,019	55.27	0.87 (0.86–0.88)
	Total	1,625,361/ 2,790,041	58.26	

	P for trend		<0.01	
≥2	Q1 (<179.00 ×10^9/L)	4,797/ 10,199	47.03	0.96 (0.90–1.01)
	Q2 (179.00–214.99 ×10^9/L)	4,959/ 10,343	47.95	1.04 (0.98–1.10)
	Q3 (215.00–246.99 ×10^9/L)	4,948/ 10,429	47.44	Ref
	Q4 (247.00–286.99 ×10^9/L)	4,724/ 10,301	45.86	0.99 (0.93–1.05)
	Q5 (≥287.00 ×10^9/L)	4,745/ 10,404	45.61	0.95 (0.90–1.00)
	Total	24,173/ 51,676	46.78	
	P for trend		0.40	
**Menstrual cycle regularity**				
Regular menstruation	Q1 (<177.00 ×10^9/L)	699,337/ 1,026,742	68.11	1.05 (1.04–1.06)
	Q2 (177.00–207.99 ×10^9/L)	702,068/ 1,023,806	68.57	1.04 (1.03–1.05)
	Q3 (208.00–235.99 ×10^9/L)	688,173/ 1,020,823	67.41	Ref
	Q4 (236.00–271.99 ×10^9/L)	684,262/ 1,037,945	65.92	0.95 (0.94–0.96)
	Q5 (≥272.00 ×10^9/L)	646,078/ 1,030,833	62.68	0.87 (0.86–0.88)
	Total	3,419,918/ 5,140,149	66.53	
	P for trend		<0.01	
Irregular menstruation	Q1 (<178.00 ×10^9/L)	24,695/ 37,529	65.80	1.07 (1.04–1.10)
	Q2 (178.00–209.99 ×10^9/L)	24,759/ 38,000	65.16	1.02 (1.00–1.05)
	Q3 (210.00–238.99 ×10^9/L)	24,081/ 37,719	63.84	Ref
	Q4 (239.00–274.99 ×10^9/L)	23,384/ 37,883	61.73	0.93 (0.91–0.96)
	Q5 (≥275.00 ×10^9/L)	22,247/ 38,472	57.83	0.84 (0.82–0.87)
	Total	119,166/ 189,603	62.85	
	P for trend		<0.01	
**BMI, kg/m^2**				
Underweight (＜18.5)	Q1 (<172.00 ×10^9/L)	88,236/ 126,955	69.50	1.03 (1.01–1.04)

	Q2 (172.00–201.99 $\times 10^9$/L)	91,356/ 130,724	69.88	1.05 (1.03–1.06)
	Q3 (202.00–228.99 $\times 10^9$/L)	87,752/ 127,146	69.02	Ref
	Q4 (229.00–264.99 $\times 10^9$/L)	87,917/ 129,977	67.64	0.96 (0.94–0.97)
	Q5 (≥264.00 $\times 10^9$/L)	85,561/ 130,379	65.62	0.90 (0.89–0.91)
	Total	440,822/ 645,181	68.33	
	P for trend		<0.01	
Normal (18.5–23.9)	Q1 (<175.00 $\times 10^9$/L)	492,378/ 721,263	68.27	1.03 (1.02–1.04)
	Q2 (175.00–205.99 $\times 10^9$/L)	516,797/ 747,312	69.15	1.04 (1.03–1.05)
	Q3 (206.00–233.99 $\times 10^9$/L)	510,202/ 748,697	68.15	Ref
	Q4 (234.00–268.99 $\times 10^9$/L)	494,153/ 740,209	66.76	0.96 (0.95–0.97)
	Q5 (≥269.00 $\times 10^9$/L)	482,784/ 749,694	64.40	0.89 (0.88–0.90)
	Total	2,496,314/ 3,707,175	67.34	
	P for trend		<0.01	
Overweight (24.0–27.9)	Q1 (<184.00 $\times 10^9$/L)	98,729/ 151,760	65.06	1.12 (1.10–1.13)
	Q2 (184.00–215.99 $\times 10^9$/L)	98,272/ 151,006	65.08	1.08 (1.07–1.10)
	Q3 (216.00–246.99 $\times 10^9$/L)	98,091/ 156,438	62.70	Ref
	Q4 (247.00–283.99 $\times 10^9$/L)	94,535/ 154,357	61.24	0.95 (0.93–0.96)
	Q5 (≥284.00 $\times 10^9$/L)	88,828/ 155,139	57.26	0.86 (0.85–0.87)
	Total	478,455/ 768,700	62.24	
	P for trend		<0.01	
Obesity (≥28.0)	Q1 (<193.00 $\times 10^9$/L)	24,896/ 39,120	63.64	1.19 (1.16–1.22)
	Q2 (193.00–226.99 $\times 10^9$/L)	24,331/ 39,108	62.21	1.09 (1.06–1.11)
	Q3 (227.00–259.99 $\times 10^9$/L)	23,808/ 40,284	59.10	Ref
	Q4 (260.00–297.99 $\times 10^9$/L)	22,442/ 39,478	56.85	0.92 (0.90–0.95)
	Q5 (≥298.00 $\times 10^9$/L)	20,872/ 40,002	52.18	0.81 (0.79–0.83)

	Total	116,349/ 197,992	58.76	
	P for trend		<0.01	
**Hypertension**				
No	Q1 (<176.00 ×10^9/L)	690,480/ 1,011,972	68.23	1.04 (1.03–1.05)
	Q2 (176.00–207.99 ×10^9/L)	733,923/ 1,067,610	68.74	1.04 (1.03–1.05)
	Q3 (208.00–235.99 ×10^9/L)	698,465/ 1,033,809	67.56	Ref
	Q4 (236.00–270.99 ×10^9/L)	678,435/ 1,026,615	66.08	0.95 (0.94–0.96)
	Q5 (≥271.00 ×10^9/L)	667,692/ 1,060,693	62.95	0.86 (0.85–0.87)
	Total	3468,995/ 5,200,699	66.70	
	P for trend		<0.01	
Yes	Q1 (<184.00 ×10^9/L)	11,112/ 20,845	53.31	1.08 (1.04–1.12)
	Q2 (184.00–217.99 ×10^9/L)	11,048/ 20,546	53.77	1.06 (1.02–1.10)
	Q3 (218.00–250.99 ×10^9/L)	11,343/ 21,461	52.85	Ref
	Q4 (251.00–289.99 ×10^9/L)	10,746/ 20,720	51.86	0.98 (0.94–1.02)
	Q5 (≥290.00 ×10^9/L)	10,316/ 21,318	48.39	0.91 (0.87–0.94)
	Total	54,565/ 104,890	52.02	
	P for trend		<0.01	
**Blood glucose level**				
Normal	Q1 (<176.00 ×10^9/L)	676,456/ 991,672	68.21	1.04 (1.03–1.05)
	Q2 (176.00–206.99 ×10^9/L)	693,142/ 1,008,103	68.76	1.04 (1.03–1.05)
	Q3 (207.00–235.99 ×10^9/L)	705,728/ 1,044,824	67.55	Ref
	Q4 (236.00–270.99 ×10^9/L)	661,232/ 1,000,886	66.06	0.95 (0.94–0.96)
	Q5 (≥271.00 ×10^9/L)	648,256/ 1,030,941	62.88	0.87 (0.86–0.88)
	Total	3,384,814/ 507,6426	66.68	
	P for trend		<0.01	

Impaired fasting glucose	Q1 (<182.00 ×10^9/L)	19,838/ 31,551	62.88	1.07 (1.03–1.10)
	Q2 (182.00–214.99 ×10^9/L)	20,579/ 32,398	63.52	1.06 (1.03–1.09)
	Q3 (215.00–244.99 ×10^9/L)	19,774/ 32,070	61.66	Ref
	Q4 (245.00–282.99 ×10^9/L)	19,706/ 32,352	60.91	0.97 (0.94–0.99)
	Q5 (≥283.00 ×10^9/L)	18,849/ 32,689	57.66	0.89 (0.87–0.92)
	Total	98,746/ 161,060	61.31	
	P for trend		<0.01	
Diabetes	Q1 (<185.00 ×10^9/L)	7,902/ 13,135	60.16	1.05 (1.01–1.11)
	Q2 (185.00–217.99 ×10^9/L)	7,846/ 13,003	60.34	1.06 (1.01–1.10)
	Q3 (218.00–248.99 ×10^9/L)	7,622/ 13,049	58.41	Ref
	Q4 (249.00–287.99 ×10^9/L)	7,469/ 13,391	55.78	0.92 (0.88–0.97)
	Q5 (≥288.00 ×10^9/L)	6,886/ 13,182	52.24	0.86 (0.82–0.91)
	Total	37,725/ 65,760	57.37	
	P for trend		<0.01	
**Anemia**				
No	Q1 (<178.00 ×10^9/L)	563,583/ 840,504	67.05	1.05 (1.04–1.06)
	Q2 (178.00–207.99 ×10^9/L)	557,872/ 828,551	67.33	1.03 (1.02–1.04)
	Q3 (208.00–235.99 ×10^9/L)	572,678/ 861,603	66.47	Ref
	Q4 (236.00–269.99 ×10^9/L)	545,029/ 837,889	65.05	0.96 (0.95–0.97)
	Q5 (≥270.00 ×10^9/L)	542,690/ 869,866	62.39	0.89 (0.88–0.90)
	Total	2,781,852/ 4,238,413	65.63	
	P for trend		<0.01	
Yes	Q1 (<172.00 ×10^9/L)	153,456/ 215,690	71.15	1.01 (1.00–1.02)
	Q2 (172.00–204.99 ×10^9/L)	157,982/ 216,252	73.05	1.05 (1.03–1.06)
	Q3 (205.00–235.99 ×10^9/L)	154,905/ 217,723	71.15	Ref

	Q4 (236.00–277.99 $\times 10^9$/L)	152,678/ 222,306	68.68	0.93 (0.92–0.94)
	Q5 (≥278.00 $\times 10^9$/L)	137,635/ 218,312	63.05	0.82 (0.81–0.83)
	Total	756,656/ 1,090,283	69.40	
	P for trend		<0.01	
**Alcohol consumption**				
No	Q1 (<176.00 $\times 10^9$/L)	687,478/ 1,008,908	68.14	1.04 (1.03–1.05)
	Q2 (176.00–206.99 $\times 10^9$/L)	703,874/ 1,024,132	68.73	1.04 (1.03–1.05)
	Q3 (207.00–235.99 $\times 10^9$/L)	717,070/ 1,062,065	67.52	Ref
	Q4 (236.00–270.99 $\times 10^9$/L)	672,066/ 1,018,185	66.01	0.95 (0.94–0.96)
	Q5 (≥271.00 $\times 10^9$/L)	663,232/ 1,056,540	62.77	0.87 (0.86–0.88)
	Total	3443,720/ 5,169,830	66.61	
	P for trend		<0.01	
Yes	Q1 (<185.00 $\times 10^9$/L)	17,300/ 28,746	60.18	1.06 (1.02–1.09)
	Q2 (185.00–216.99 $\times 10^9$/L)	17,884/ 29,610	60.40	1.05 (1.03–1.10)
	Q3 (217.00–245.99 $\times 10^9$/L)	17,462/ 29,559	59.08	Ref
	Q4 (246.00–281.99 $\times 10^9$/L)	17,219/ 29,405	58.56	0.98 (0.95–1.02)
	Q5 (≥282.00 $\times 10^9$/L)	16,527/ 29,632	55.77	0.89 (0.91–0.97)
	Total	86,392/ 146,952	58.79	
	P for trend		<0.01	
**Tobacco exposure**				
No	Q1 (<176.00 $\times 10^9$/L)	639,061/ 934,159	68.41	1.04 (1.03–1.05)
	Q2 (176.00–206.99 $\times 10^9$/L)	651,560/ 945,973	68.88	1.04 (1.03–1.05)
	Q3 (207.00–235.99 $\times 10^9$/L)	663,416/ 980,928	67.63	Ref
	Q4 (236.00–270.99 $\times 10^9$/L)	620,907/ 939,271	66.11	0.95 (0.94–0.96)
	Q5 (≥271.00 $\times 10^9$/L)	613,353/ 975,201	62.90	0.86 (0.85–0.87)

	Total	3,188,297/ 4,775,532	66.76	
	P for trend		<0.01	
Yes	Q1 (<180.00 $\times 10^9$/L)	69,031/ 107,990	63.92	1.04 (1.02–1.05)
	Q2 (180.00–209.99 $\times 10^9$/L)	67,803/ 103,895	65.26	1.04 (1.02–1.06)
	Q3 (210.00–238.99 $\times 10^9$/L)	70,741/ 110,291	64.14	Ref
	Q4 (239.00–274.99 $\times 10^9$/L)	68,664/ 109,177	62.89	0.96 (0.94–0.97)
	Q5 (≥275.00 $\times 10^9$/L)	64,864/ 108,881	59.57	0.90 (0.89–0.92)
	Total	341,103/ 540,234	63.14	
	P for trend		<0.01	
**Contraceptive measures used before**				
No	Q1 (<176.00 $\times 10^9$/L)	491547/ 666743	73.72	1.05 (1.04–1.06)
	Q2 (176.00–206.99 $\times 10^9$/L)	501154/ 678705	73.84	1.04 (1.03–1.05)
	Q3 (207.00–234.99 $\times 10^9$/L)	489649/ 675781	72.46	Ref
	Q4 (235.00–270.99 $\times 10^9$/L)	488405/ 691327	70.65	0.95 (0.94–0.96)
	Q5 (≥271.00 $\times 10^9$/L)	455011/ 678394	67.07	0.86 (0.85–0.87)
	Total	2,425,766/ 3,390,950	71.54	
	P for trend		<0.01	
Yes	Q1 (<178.00 $\times 10^9$/L)	220220/ 382998	57.50	1.03 (1.02–1.04)
	Q2 (178.00–208.99 $\times 10^9$/L)	221963/ 377967	58.73	1.03 (1.02–1.04)
	Q3 (209.00–237.99 $\times 10^9$/L)	227249/ 390681	58.17	Ref
	Q4 (238.00–273.99 $\times 10^9$/L)	217707/ 381038	57.14	0.96 (0.95–0.97)
	Q5 (≥274.00 $\times 10^9$/L)	213913/ 388754	55.03	0.91 (0.90–0.92)
	Total	1,101,052/ 1,921,438	57.30	
	P for trend		<0.01	

**Gynecological abnormalities**				
No	Q1 (<176.00 ×10^9/L)	326263/ 499743	65.29	1.04 (1.03–1.05)
	Q2 (176.00–207.99 ×10^9/L)	337694/ 513506	65.76	1.04 (1.03–1.05)
	Q3 (208.00–235.99 ×10^9/L)	324389/ 502298	64.58	Ref
	Q4 (236.00–271.99 ×10^9/L)	325631/ 514756	63.26	0.95 (0.94–0.96)
	Q5 (⩾272.00 ×10^9/L)	313180/ 519811	60.25	0.87 (0.86–0.88)
	Total	1627157/ 2550114	63.81	
	P for trend		<0.01	
Yes	Q1 (<185.00 ×10^9/L)	4,755/ 11,190	42.49	1.05 (1.01–1.09)
	Q2 (185.00–217.99 ×10^9/L)	5,192/ 11,444	45.37	1.04 (0.99–1.08)
	Q3 (218.00–248.99 ×10^9/L)	3,147/ 6,967	45.17	Ref
	Q4 (249.00–273.99 ×10^9/L)	2,118/ 4,771	44.39	0.99 (0.95–1.04)
	Q5 (⩾286.00 ×10^9/L)	4,872/ 23,076	43.22	0.93 (0.89–0.97)
	Total	25,185/ 57,448	43.84	
	P for trend		<0.01	

CI, confidence interval; FR, fecundability ratio; Q, quintile.

[a] FRs were adjusted for women's demographic characteristics (women's age (continuous), husband's age (continuous), region, ethnicity, educational level, occupation, pregnancy history, number of children in the current family, age at menarche (continuous), menstrual cycle regularity) and women's health status and lifestyles (body mass index (continuous), hypertension, fasting plasma glucose level, hemoglobin (continuous), alcohol consumption, tobacco exposure, contraceptive measures used before, and gynecological abnormalities).

eTable 3. The fecundability ratios for women with regular menstruation that calculated by TTP in cycles

Categories	Pregnancies/ N	Pregnancy rate (%)	Crude FR (95% CI)	Adjusted FR (95% CI) [a]	Adjusted FR (95% CI) [b]
Q1 (<177.00 ×10^9/L)	699,337/ 1,026,742	68.11	1.03 (1.02–1.04)	1.04 (1.03–1.05)	1.04 (1.03–1.05)
Q2 (177.00–207.99 ×10^9/L)	702,068/ 1,023,806	68.57	1.04 (1.03–1.05)	1.04 (1.03–1.05)	1.03 (1.02–1.04)
Q3 (208.00–235.99 ×10^9/L)	688,173/ 1,020,823	67.41	Ref	Ref	Ref
Q4 (236.00–271.99 ×10^9/L)	684,262/ 1,037,945	65.92	0.94 (0.93–0.95)	0.95 (0.94–0.96)	0.95 (0.94–0.96)
Q5 (≥272.00 ×10^9/L)	646,078/ 1,030,833	62.68	0.86 (0.85–0.87)	0.87 (0.86–0.88)	0.87 (0.86–0.88)
P for trend					<0.01

CI, confidence interval; FR, fecundability ratio; TTP, time to pregnancy.

[a] FRs were adjusted for women's demographic characteristics (women's age (continuous), husbands' age (continuous), region, ethnicity, educational level, occupation, pregnancy history, number of children in the current family, age at menarche (continuous)).

[b] FRs were additionally adjusted for women's health status and lifestyles (body mass index (continuous), hypertension, blood glucose level, hemoglobin (continuous), alcohol consumption, tobacco exposure, contraceptive measures used before, and gynecological abnormalities).

eTable 4. Sensitivity analysis for the association between platelet count and fecundability (based on the imputed data after multivariate imputation)

Categories	Pregnancies/ N	Pregnancy rate (%)	Adjusted FR (95% CI) [a]
Q1 (<177.00 ×10^9/L)	723,366/ 1,063,257	68.03	1.04 (1.03–1.05)
Q2 (177.00–207.99 ×10^9/L)	725,852/ 1,060,250	68.46	1.04 (1.03–1.05)
Q3 (208.00–235.99 ×10^9/L)	711,578/ 1,057,408	67.29	Ref
Q4 (236.00–271.99 ×10^9/L)	708,481/ 1,077,016	65.78	0.95 (0.94–0.96)
Q5 (≥272.00 ×10^9/L)	669,807/ 1,071,821	62.49	0.86 (0.85–0.87)
P for trend			<0.01

CI, confidence interval; FR, fecundability ratio; Q, quintile.

[a] FRs were adjusted for women's demographic characteristics (women's age (continuous), husband's age (continuous), region, ethnicity, educational level, occupation, pregnancy history, number of children in the current family, age at menarche (continuous), menstrual cycle regularity) and women's health status and lifestyles (body mass index (continuous), hypertension, fasting plasma glucose level, hemoglobin (continuous), alcohol consumption, tobacco exposure, contraceptive measures used before, and gynecological abnormalities).

eTable 5. Sensitivity analysis for the association between platelet count and fecundability (excluding the women whose PLT<100×10^9/L or PLT>400×10^9/L)

**Categories**	**Pregnancies/ N**	**Pregnancy rate (%)**	**Adjusted FOR (95% CI)** [a]
Q1 (<178.00 ×10^9/L)	713,848/ 1,045,016	68.31	1.05 (1.04–1.06)
Q2 (178.00–207.99 ×10^9/L)	707,698/ 1,033,770	68.46	1.03 (1.02–1.04)
Q3 (208.00–235.99 ×10^9/L)	711,578/ 1,057,408	67.29	Ref
Q4 (236.00–270.99 ×10^9/L)	692,388/ 1,052,408	65.79	0.95 (0.94–0.96)
Q5 (≥271.00 ×10^9/L)	663,243/ 1,056,574	62.77	0.88 (0.87–0.89)
P for trend			<0.01

CI, confidence interval; FR, fecundability ratio; Q, quintile.

[a] FRs were adjusted for women's demographic characteristics (women's age (continuous), husband's age (continuous), region, ethnicity, educational level, occupation, pregnancy history, number of children in the current family, age at menarche (continuous), menstrual cycle regularity) and women's health status and lifestyles (body mass index (continuous), hypertension, fasting plasma glucose level, hemoglobin (continuous), alcohol consumption, tobacco exposure, contraceptive measures used before, and gynecological abnormalities).

eFigure 1. Kaplan-Meier plot for pre-pregnancy maternal platelet count status (n=5,329,752)

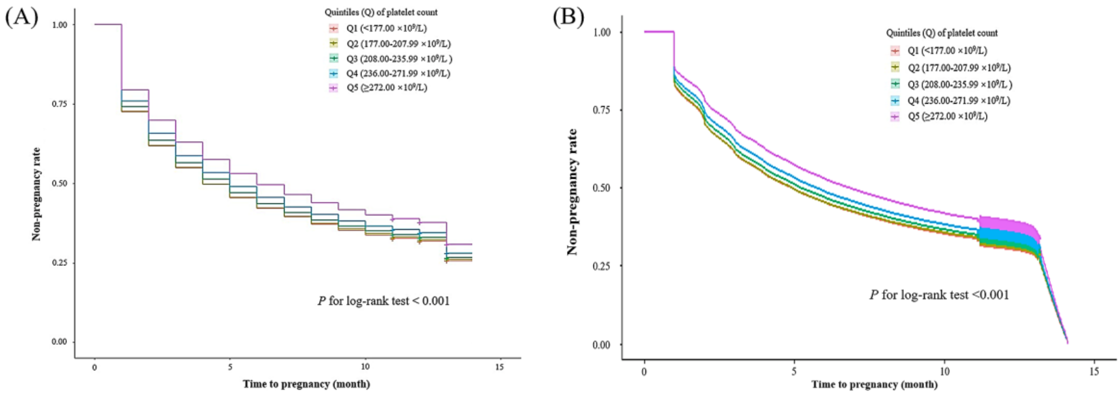

(**A**) The Cox regression models for discrete survival time were adopted (TTP in months rounded down); (**B**) The Cox proportional hazards regression models were adopted (TTP in months not rounded down).

eFigure 2. The association between platelet count and fecundability stratified by pregnant history with the use of restricted cubic splines

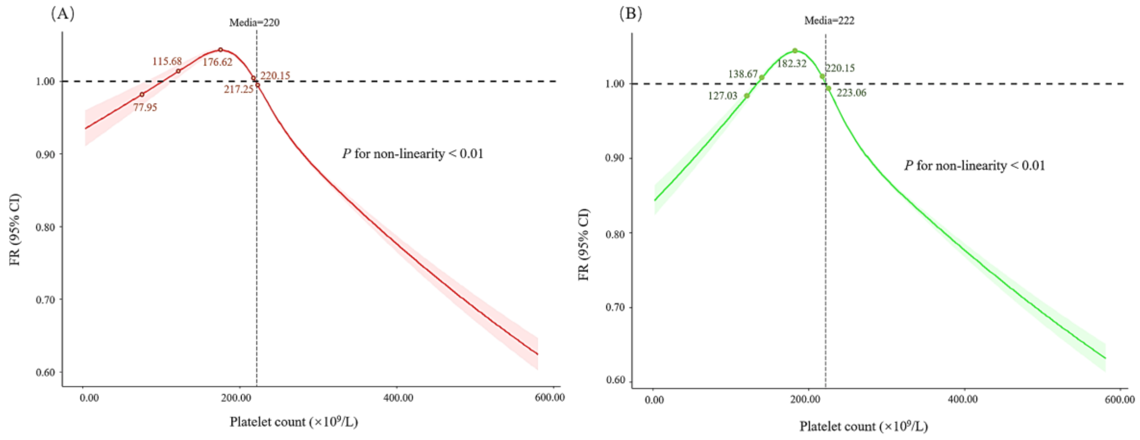

(**A**) Women without pregnant history; (**B**) Women with pregnant history. There are 4 knots located at the 5th, 35th, 65th and 95th percentiles of the distribution of platelet count. Dashed lines correspond to reference values. Shaded areas represent 95% confidence intervals for FR. The median platelet count was used as a reference. CI, confidence interval; FR, fecundability ratio. *FRs were adjusted for women's demographic characteristics (women's age (continuous), husband's age (continuous), region, ethnicity, educational level, occupation, number of children in current family, age at menarche (continuous), menstrual cycle regularity) and female partners' healthy status and lifestyles (body mass index (continuous), hypertension, fasting plasma glucose level, hemoglobin (continuous), alcohol consumption, tobacco exposure, contraceptive measures used before, and gynecological abnormalities).

eFigure 3. The association between platelet count and fecundability stratified by menstrual cycle regularity with the use of restricted cubic splines

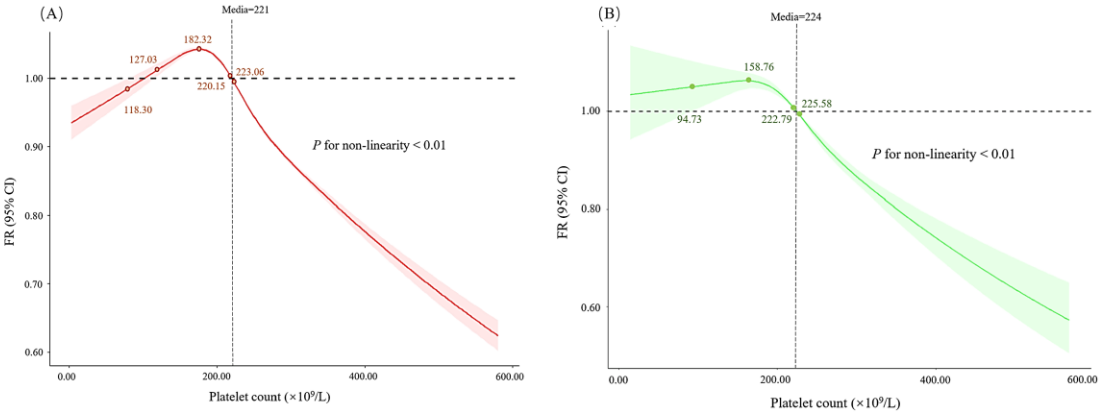

(**A**) Women with regular menstruation; (**B**) Women with irregular menstruation. There are 4 knots located at the 5th, 35th, 65th and 95th percentiles of the distribution of platelet count. Dashed lines correspond to reference values; Shaded areas represent 95% confidence intervals for FR. The median platelet count was used as a reference. CI, confidence interval; FR, fecundability odds ratio. *FRs were adjusted for women's demographic characteristics (women's age (continuous), husband's age (continuous), region, ethnicity, educational level, occupation, pregnancy history, number of children in current family, age at menarche (continuous)) and female partners' healthy status and lifestyles (body mass index (continuous), hypertension, fasting plasma glucose level, hemoglobin (continuous), alcohol consumption, tobacco exposure, contraceptive measures used before, and gynecological abnormalities).
